# The effect of brain serotonin deficiency on breathing is magnified by age

**DOI:** 10.14814/phy2.15245

**Published:** 2022-05-17

**Authors:** Huy Pho, Mateus R. Amorim, Qingchao Qiu, Mi‐Kyung Shin, Lenise J. Kim, Frederick Anokye‐Danso, Jonathan J. Jun, Rexford S. Ahima, Luiz G. S. Branco, Donald M. Kuhn, Jason H. Mateika, Vsevolod Y. Polotsky

**Affiliations:** ^1^ Division of Pulmonary and Critical Care Medicine Department of Medicine Johns Hopkins University School of Medicine Baltimore Maryland USA; ^2^ Department of Physiology Wayne State University Detroit Michigan USA; ^3^ Division of Endocrinology, Diabetes, and Metabolism Department of Medicine Johns Hopkins University School of Medicine Baltimore Maryland USA; ^4^ Dental School of Ribeirão Preto University of São Paulo São Paulo Brazil; ^5^ Department of Psychiatry and Behavioral Neurosciences Wayne State University School of Medicine Detroit Michigan USA; ^6^ John D. Dingell Veterans Affairs Medical Center Detroit Michigan USA; ^7^ Department of Internal Medicine Wayne State University School of Medicine Detroit Michigan USA

**Keywords:** aging, breathing, serotonin, sleep

## Abstract

Serotonin is an important mediator modulating behavior, metabolism, sleep, control of breathing, and upper airway function, but the role of aging in serotonin‐mediated effects has not been previously defined. Our study aimed to examine the effect of brain serotonin deficiency on breathing during sleep and metabolism in younger and older mice. We measured breathing during sleep, hypercapnic ventilatory response (HCVR), CO_2_ production (VCO_2_), and O_2_ consumption (VO_2_) in 16–18‐week old and 40–44‐week old mice with deficiency of tryptophan hydroxylase 2 (*Tph2*), which regulates serotonin synthesis specifically in neurons, compared to *Tph2* ^+/+^ mice. As expected, aging decreased VCO_2_ and VO_2_. *Tph2* knockout resulted in an increase in both metabolic indexes and no interaction between age and the genotype was observed. During wakefulness, neither age nor genotype had an effect on minute ventilation. The genotype did not affect hypercapnic sensitivity in younger mice. During sleep, *Tph2* ^−/−^ mice showed significant decreases in maximal inspiratory flow in NREM sleep, respiratory rate, and oxyhemoglobin saturation in REM sleep, compared to wildtype, regardless of age. Neither serotonin deficiency nor aging affected the frequency of flow limited breaths (a marker of upper airway closure) or apneas. Serotonin deficiency increased the amount and efficiency of sleep only in older animals. In conclusion, younger *Tph2* ^−/−^ mice were able to defend their ventilation and phenotypically did not differ from wildtype during wakefulness. In contrast, both young and old *Tph2* ^−/−^ mice showed sleep‐related hypoventilation, which was manifested by hypoxemia during REM sleep.

## Graphical abstract

To our knowledge, this is the first study, which examined the effect of neuronal serotonin deficiency on a combination of metabolic and respiratory parameters across ages. Aging greatly magnified the effects of brain serotonin deficiency on breathing and on hypercapnic sensitivity. Neuronal serotonin deficiency increased total sleep time and the amount of REM sleep.

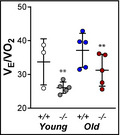

## 1 INTRODUCTION

Serotonin is a ubiquitous neuro‐modulator–transmitter regulating sleep (Portas et al., 2000), metabolism (Watanabe et al., 2010), control of breathing, and upper airway function (Hilaire et al., 2010). Serotonin is a product of hydroxylation of l‐tryptophan, and two different isoforms of l‐tryptophan hydroxylase (TPH) regulate serotonin biosynthesis in the brain and at the periphery. TPH2 controls neuronal serotonin biosynthesis. Serotonin acts in the brain to decrease metabolic rate and to enhance CO_2_ responses (Cummings & Hodges, 2019; Hilaire et al., 2010; Hodges & Richerson, 2008a, 2010; Hodges et al., 2008) and pharyngeal muscle activity (Fenik et al., 2005; Kubin et al., 1996, 1998; Kubin & Volgin, 2008). We have previously shown that *Tph2* ^−/−^ mice, whose brain is depleted of serotonin (Alenina et al., 2009), have altered sleep architecture, increased metabolic rate (Hickner et al., 2014), increased motor activity (Solarewicz et al., 2015) and apnea frequency (Mateika et al., 2019).

However, many questions remain unanswered. First, it is unknown if decreased CO_2_ responses in serotonin deficiency result in hypoventilation during sleep. Second, the effect of brain serotonin deficiency on upper airway patency during sleep has not been elucidated. Third, the role of aging in respiratory and metabolic responses to brain serotonin deficiency has not been studied.

The goal of this study was to examine the effect of brain serotonin deficiency on breathing during sleep, the hypercapnic ventilatory response (HCVR), and CO_2_ production (VCO_2_) in 16–18‐ week‐old and 40–44‐week‐old *Tph2* ^−/−^ and *Tph2* ^+/+^ mice. We hypothesized that brain serotonin deficiency affects control of breathing and airway patency during sleep and that these responses are magnified by age. To test our hypothesis we employed our plethysmographic methods for monitoring high‐fidelity airflow and respiratory effort signals continuously during sleep (Hernandez et al., 2012). We defined upper airway obstruction as the presence of inspiratory airflow limitation characterized by an early inspiratory plateau in airflow at a maximum level (*V*_I_max), whereas effort continued to increase (Condos et al., 1994; Fleury Curado et al., 2018; Gold & Schwartz, 1996; Hernandez et al., 2012; Schwartz et al., 1988; Schwartz, Brower, et al., 1989; Schwartz, Smith, et al., 1989).

## 2 METHODS

### 2.1 Animals

*Tph2* ^+/+^ and *Tph2* ^−/−^ male mice were generated in Dr. Donald Kuhn's laboratory at Wayne State University (Thomas et al., 2010). In total, 27 mice (Younger—*Tph2* ^+/+^ [*n* = 7], *Tph2* ^−/−^ [*n* = 5]; Older—*Tph2* ^+/+^ [*n* = 8], *Tph2* ^−/−^ [*n* = 7]) were used in the present study. Water and food were available *ad libitum*. Mice were housed at a 12‐h light/dark cycle (7 am–7 pm lights on) and temperature of ~30°C. Food consumption and body weight were monitored daily throughout the sleep study protocol. Following completion of all protocols, mice were euthanized by anesthetic overdose and dislocation of the neck. All protocols were approved by the Johns Hopkins University Animal Care and Use Committee (ACUC, Protocol #MO19M191) and all animal experiments were conducted in accordance with ACUC guidelines.

### 2.2 Sleep studies

Polysomnography was performed in young and old *Tph2* ^+/+^ and *Tph2* ^−/−^ mice. Mice were anesthetized with isoflurane 1%–2% and placed in the stereotaxic system (Model 963 with 923‐B Head Holder, David Kopf Instruments) where a headmount procedure was performed immediately. The headmount (no. 8201, Pinnacle Technology) was implanted for electroencephalogram (EEG) and electromyogram (EMG) recordings. Briefly, four holes were bored through the skull in frontal and parietal region to allow implantation of electrode screws. EMG leads were tunneled subcutaneously and placed over the nuchal muscle posterior to the skull. Dental acrylic (Lang Dental) was used to secure the headmount in place. Immediately after the surgeries, all mice received 0.03 mg/kg of Buprenorphine intraperitoneally and were housed in a recovery chamber under a heating lamp. Mice were monitored and received additional 0.03 mg/kg of Buprenorphine if signs of distress or pain were observed. Animals were studied 2 weeks post headmount implantation.

For polysomnography we used a modified whole‐body plethysmography (WBP, EMMS) chamber system to measure tidal airflow and sleep‐wake state continuously, generating high‐fidelity tidal volume and airflow signals, as previously described (Fleury Curado et al., 2018; Hernandez et al., 2012; Pho et al., 2021). In brief, mice were acclimated to the chamber prior to sleep study recording. Mice were weighed, injected with normal saline (1 ml) subcutaneously and placed in the WBP chamber to be recorded from 10.00 am to 4.00 pm on the day of the study. Mouse weight was recorded prior to being placed in the chamber. Animals fasted during the study. Mouse rectal temperature was measured at the beginning and end of the sleep study. During full polysomnographic recordings, the chamber was humidified to ~90% relative humidity and ~30°C while a slow leak allowed atmospheric pressure equilibrium. The WBP’s reference chamber filtered out ambient noise from the pressure signal acquired by a transducer. Positive and negative pressure sources were utilized in series with mass flow controllers (Alicat Scientific) and high‐resistance elements to generate a continuous bias airflow through the animal chamber while maintaining a sufficiently high time constant. Tidal airflow was calculated from the plethysmography chamber pressure signal using the Drorbaugh and Fenn equation (Drorbaugh & Fenn, 1955) which required the measurements of mouse rectal temperature, chamber temperature, room temperature, relative humidity, and chamber gas constant, calculated by utilizing the chamber pressure deflection of a known volume injection. The tidal volume signal was differentiated electronically to generate an airflow signal. Oxyhemoglobin saturation (SpO_2_) was measured using an oxygen sensor collar from the MouseOxPlus system (Starr Life Sciences).

All signals were digitized at 1000 Hz (sampling frequency per channel) and recorded in LabChart 7 Pro (Version 7.2, ADInstruments). Sleep‐wake state was scored visually in 5 s epochs based off standard criteria of EEG and EMG frequency content and amplitude, as previously described (Amorim et al., 2021; Fleury Curado et al., 2018; Pho et al., 2016, 2021; Yao et al., 2016). Wakefulness was characterized by low‐amplitude, high‐frequency (~10 to 20 Hz) EEG waves and high levels of EMG activity compared with the sleep states. NREM sleep was characterized by high‐amplitude, low frequency (~2 to 5 Hz) EEG waves with EMG activity considerably less than during wakefulness. REM sleep was characterized by low‐amplitude, mixed frequency (~5 to 10 Hz) EEG waves with EMG amplitude either below or equal to that during NREM sleep. Sleep efficiency was calculated as the amount of sleep divided by the total recording time after sleep onset and reported as a percentage. Respiratory signals were analyzed from all REM sleep periods and from periods of NREM sleep sampled periodically at 20‐s stretches every half an hour throughout the total recording time. Custom software was used to demarcate the start and end of inspiration and expiration for subsequent calculations of timing and amplitude parameters for each respiratory cycle.

We utilized each breath's respiratory characteristic to describe maximal inspiratory airflow (*V*_I_max) and components of minute ventilation (*V*_E_). We developed an algorithm using the airflow and respiratory effort signals to determine if a breath was classified as inspiratory airflow limited, defined by an early inspiratory plateau in airflow while effort continued to increase. The software provided peak flow values during the first half (*V*_I_max1), midpoint (*V*_I_50), and second half (*V*_I_max2) of inspiration. Breaths resembling sniffs were initially defined as non‐flow limited by their short duration, having an inspiration time with a *z*‐score lower than 1.75. Breaths having sufficient inspiration time were then classified as inspiratory flow limited if a mid‐inspiratory flow plateau was present (Fleury Curado et al., 2018; Pho et al., 2016; Yao et al., 2016).

### 2.3 Metabolic measurements

Metabolic studies were performed in a subset of mice (*n* = 20, 5 from each group, younger *Tph2^+/+^*, younger *Tph2* ^−/−^, older *Tph2^+/+^*, older *Tph2* ^−/−^) as previously described (Pho et al., 2021). Mice were placed in individual Comprehensive Laboratory Animal Monitoring System (CLAMS) units (Oxymax series; Columbus Instruments) for a 24‐hacclimation period followed by 24 h of continuous recordings starting at 10:00 am. The CLAMS units were sealed and equipped with O_2_ electrochemical sensors, CO_2_ infrared sensors and infrared beam movement sensors. Consumed O_2_ (*V*O_2_) and produced CO_2_ (VCO_2_) were collected every 11 min and measurements were utilized to calculate the respiratory exchange ratio (RER). Motor activity was quantified by the number of infrared beam interruptions. Total horizontal and vertical beam breaks were summed and presented as motor activity. Metabolic cages were kept in a 12‐h light/dark cycle (7 am–7 pm lights on) with food and water *ad libitum* and a consistent environmental temperature of 30°C.

### 2.4 Hypercapnic ventilatory response

Hypercapnic ventilatory response (HCVR) measurements were performed at thermoneutral conditions (30°C) in a neonatal incubator (Draeger 8000 IC), which has been adapted for respiratory measurements (Polotsky et al., 2001). HCVR was measured during the light phase while mice were awake. Mice were exposed to a gas mixture of 8% of CO_2_, 21% of O_2_, and balanced in nitrogen. Mice were acclimated with a continuous bias flow controlled with mass flow controllers at room air for 20 min. For exposure, room air was switch to the hypercapnic mixture, and analyses were done after 1 min of exposure when the ventilation reached a plateau. Tidal volume (*V*_T_), respiratory rate (RR), and minute ventilation (*V*_E_) were measured in young and old *Tph2* ^+/+^ and *Tph2^−/−^* mice. HCVR was determined in each animal by the slope of the relationship between minute ventilation (*V*_E_) and inspired CO_2_ (0%–8%) during wakefulness via linear least‐squares regression analysis.

### 2.5 Apnea scoring

Apnea scoring was done in accordance with our previous study (Freire et al., 2020; Kim et al., 2021). Apneas were scored as ≥90% reduction in airflow for a period corresponding to two or more breath cycles or ≥0.7 s based on average respiratory rate at baseline.

### 2.6 Statistical Analysis

Data were tested for normality using Shapiro–Wilk's test. All the data of the present study followed normality and were analyzed by two‐way ANOVA with the Tukey’s *post hoc* test. Statistical analyses were performed in using Prism, version 7.03 (GraphPad Software Inc.) and the data are represented as mean ± SD. Statistical significance was considered at a level of *p* < 0.05, although the exact *p* values are reported.

## 3 RESULTS

### 3.1 Baseline characteristics of *Tph2^+/+^* and *Tph2* ^−/−^ mice

Compared to *Tph2^+/+^* mice, *Tph2^−/−^* mice showed significant increases in body temperature (younger: *p* = 0.025; older: *p* = 0.0094) in both age groups, whereas food intake was significantly increased only in older mice (*p* = 0.0030) and body weight was significantly reduced only in older mice (*p* = 0.0004) (Table 1).

**TABLE 1:** Characteristics of age, weight, temperature, and food intake of *Tph2* ^−/−^ and *Tph2* ^+/+^ mice. Younger—*Tph2* ^+/+^: (*n* = 7), *Tph2* ^−/−^: (*n* = 5); Older—*Tph2* ^+/+^ (*n* = 8), *Tph2* ^−/−^ (*n* = 7)

	*n*	Age (weeks)	*p* value	Weight (g)	*p* value	*T* (°C)	*p* value	Food intake (g)	*p* value
Young									
*Tph2^+/+^*	7	18.2 ± 0.9	—	27.4 ± 1.2	—	35.6 ± 0.5	—	3.2 ± 0.2	—
*Tph2^−/−^*	5	17.1 ± 1.7	0.9941	25.1 ± 2.9	0.2254	36.6 ± 0.4	0.025	3.6 ± 0.3	0.2335
Old									
*Tph2^+/+^*	8	39.9 ± 8.9	—	31.4 ± 2.2	—	35.4 ± 0.7	—	3.6 ± 0.3	—
*Tph2^−/−^*	7	44.6 ± 12.2	0.6561	26.3 ± 1.5	0.0004	36.4 ± 0.4	0.0094	4.5 ± 0.6	0.003

### 3.2 The effect of *Tph*2 deficiency on sleep architecture

Compared to *Tph2^+/+^* mice, older *Tph2^−/−^* mice showed significant increases in sleep efficiency (*p* = 0.0043), total sleep time (*p* = 0.0033) and NREM sleep time (*p* = 0.0048), whereas the duration of NREM bouts was increased in both age groups (younger: *p* = 0.0219; older: *p* = 0.0045, Table 2). In addition, *Tph2* deficiency significantly extended REM sleep in both age groups (younger: *p* = 0.0188; older: *p* = 0.0140), increasing the number of bouts in younger mice (*p* = 0.0374), and the bout length in older mice (*p* = 0.0045).

**TABLE 2:** Sleep architecture of *Tph2* ^−/−^ and *Tph2* ^+/+^ mice. Younger – *Tph2* ^+/+^ (*n* = 5), *Tph2* ^−/−^ (*n* = 5); Older – *Tph2* ^+/+^ (*n* = 8), *Tph2* ^−/−^ (*n* = 7)

	Sleep efficiency (%)	*p* value	Sleep (min)						Number				Average length (min)			
			Total	*p* value	NREM	*p* value	REM	*p* value	NREM	*p* value	REM	*p* value	NREM	*p* value	REM	*p* value
Young																
*Tph2* ^+/+^	46.0 ± 9.4	—	142.8 ± 28.9	—	132.2 ± 28.7	—	10.6 ± 3.9	—	42.2 ± 10.0	—	5.8 ± 2.2	—	2.8 ± 0.4	—	1.9 ± 0.4	—
*Tph2* ^−/−^	60.0 ± 5.5	0.1894	200.9 ± 30.12	0.1529	178.8 ± 26.7	0.2486	22.1 ± 4.3	0.0188	27.0 ± 5.1	0.649	10.6 ± 0.9	0.0374	4.8 ± 1.1	0.0219	2.1 ± 0.3	0.6731
Old																
*Tph2* ^+/+^	44.6 ± 13.7	—	156.2 ± 48.82	—	145.3 ± 45.4	—	11.0 ± 4.6	—	53.1 ± 30.0	—	7.6 ± 2.3	—	2.8 ± 1.2	—	1.4 ± 0.3	—
*Tph2* ^−/−^	66.0 ± 9.6	0.0043	242.3 ± 45.82	0.0033	221.5 ± 41.1	0.0048	20.8 ± 8.0	0.014	39.0 ± 18.0	0.5527	9.7 ± 1.4	0.4237	4.9 ± 1.0	0.0045	2.1 ± 0.2	0.0014

### 3.3 The effect of *Tph2* deficiency on breathing during sleep

We have developed a high‐fidelity system allowing to differentially assess control of breathing and upper airway function during sleep (Fleury Curado et al., 2018; Hernandez et al., 2012). Our analysis is based on highly standardized detection of inspiratory flow limitation, which is a hallmark sign of upper airway obstruction during sleep. Here, we describe the effect of brain serotonin deficiency on non‐flow limited breathing, which characterizes control of breathing, and inspiratory flow limited or obstructed breathing, which is mostly determined by upper airway patency during sleep.

**FIGURE 1:**
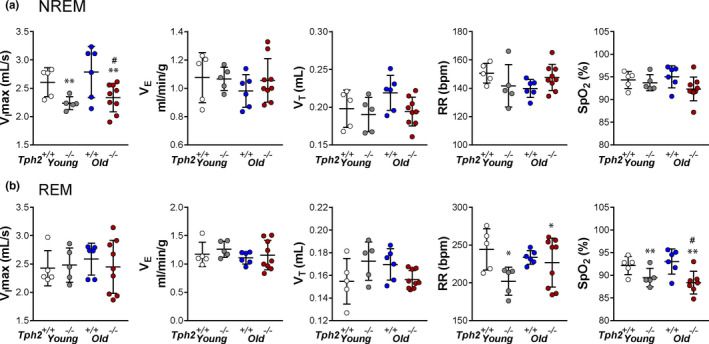
Individual and grouped data showing the age‐related differences between *Tph2* ^−/−^ and *Tph2* ^+/+^ mice on maximal inspiratory flow (*V*_I_max), minute ventilation (*V*_E_), tidal volume (*V*_T_), respiratory rate (RR), and oxygen saturation (SpO_2_) during non‐flow limited breathing in non‐rapid eye movement (NREM) (A) and rapid eye movement sleep (B). Younger—*Tph2* ^+/+^ (*n* = 5), *Tph2* ^−/−^ (*n* = 5); Older—*Tph2* ^+/+^ (*n* = 6), *Tph2* ^−/−^ (*n* = 9). *Tph*, tryptophan hydroxylase. *p < 0.05, **p < 0.01—effect of genotype using two‐way ANOVA. #*p* < 0.05 Tukey's *post hoc* test in comparison with older *Tph2* ^+/+^mice

Regardless of age, *Tph2* deficiency significantly decreased maximal airflow (*V*_I_max, *p* = 0.0028) of non‐flow‐limited breaths during NREM sleep, compared to *Tph2^+/+^* mice. A significant reduction of RR (*p* = 0.0271) and SpO_2_ (*p* = 0.0018) was also observed in *Tph2^−/−^* mice in REM sleep without effect of age (Figure 1). Within age groups analysis of non‐flow‐limited breathing showed that only older *Tph2^−/−^* mice had a marked reduction in maximal airflow (*Tph2^+/+^*: 2.8 ± 0.5 ml s^−1^ vs *Tph2^−/−^*: 2.3 ± 0.3 ml s^−1^, *p* = 0.0387) and a significant decrease in SpO_2_ (*Tph2^+/+^*: 93 ± 3% vs *Tph2^−/−^*: 88 ± 3%, *p* = 0.0120, Figure 1).

**FIGURE 2:**
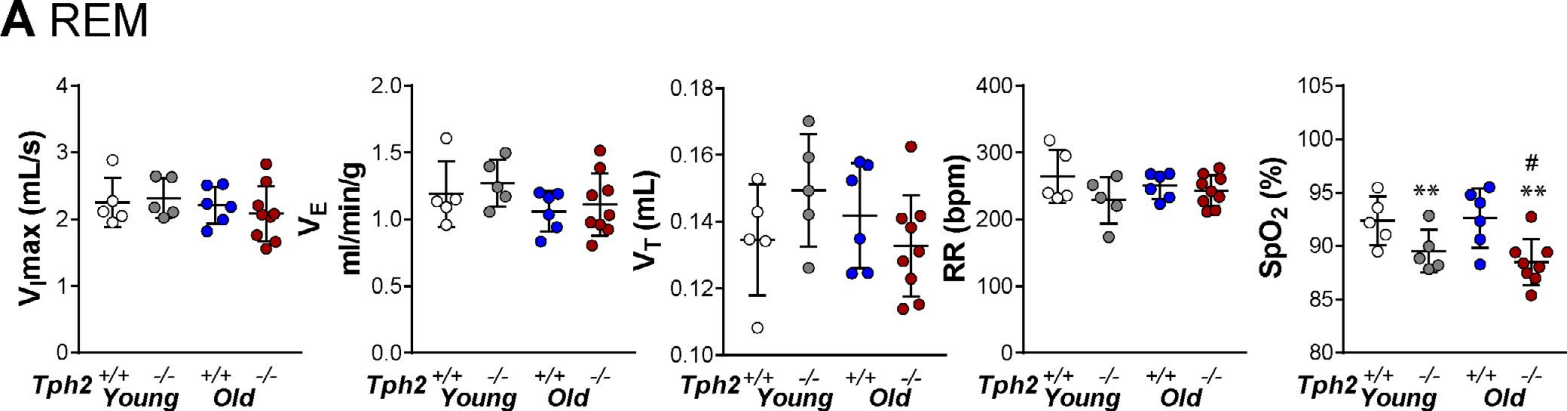
Individual and grouped data showing the age‐related differences between *Tph2* ^−/−^ and *Tph2* ^+/+^ mice on maximal inspiratory flow (*V*_I_max), minute ventilation (*V*_E_), tidal volume (*V*_T_), respiratory rate (RR), and oxygen saturation (SpO_2_) during flow limited in rapid eye movement sleep (A). Younger—*Tph2* ^+/+^ (*n* = 5), *Tph2* ^−/−^ (*n* = 5); Older—*Tph2* ^+/+^ (*n* = 6), *Tph2* ^−/−^ (*n* = 9). *Tph*, tryptophan hydroxylase. **p < 0.01—effect of genotype using two‐way ANOVA. # *p* < 0.05 Tukey's *post hoc* test in comparison with older *Tph2* ^+/+^ mice

Flow limited breaths were uncommon during NREM sleep and, therefore, were not quantified in the present study. Inspiratory flow limited breathing was prevalent in REM sleep. A deficiency in *Tph2* did not impact the frequency of obstructed breaths, regardless of age (younger—*Tph2^+/+^*: 16 ± 4% vs *Tph2^−/−^*: 12 ± 3%; older—*Tph2^+/+^*: 11 ± 4% vs *Tph2^−/−^*: 10 ± 2%, *p* = 0.2881, Figure 2). Similar to non‐flow limited breathing, *Tph2* deficiency decreased SpO_2_ in REM sleep, regardless age (*p* = 0.0018), but the comparison within groups showed that marked SpO_2_ desaturations were present predominantly in older mice (*Tph2^+/+^*: 93 ± 3% vs *Tph2^−/−^*: 88 ± 2%, *p* = 0.0181, Figure 2).

### 3.4 The effect of *Tph2* deficiency on apneas

Neither *Tph2* deficiency, aging, or a combination of the two had an effect on the frequency of apneas (younger—*Tph2^+/+^*: 17.5 ± 16.6 h^−1^ vs *Tph2^−/−^*: 14.7 ± 11.9 h^−1^; older—*Tph2^+/+^*: 26.0 ± 17.0 h^−1^ vs *Tph2^−/−^*: 11.7 ± 6.1 h^−1^, *p* = 0.3088) or the average length of apneic events (younger—*Tph2^+/+^*: 1.04 ± 0.14 s vs *Tph2^−/−^*: 1.04 ± 0.43 s; older—*Tph2^+/+^*: 0.99 ± 0.13 s vs *Tph2^−/−^*: 1.09 ± 0.23 s, *p* = 0.6201). The respiratory effort was absent during apneas indicating that they were central.

### 3.5 The effect of *Tph2* deficiency on metabolism

**FIGURE 3:**
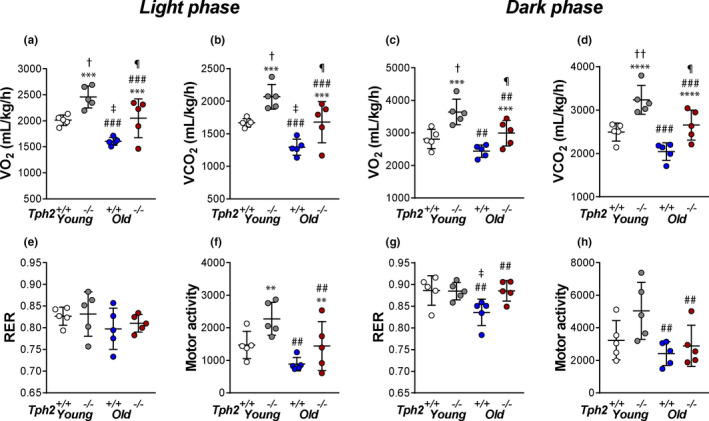
Individual and grouped data showing the age‐related differences between *Tph2* ^−/−^ and *Tph2* ^+/+^ mice on total oxygen consumption (*V*O_2_) (a), total carbon dioxide production (*V*CO_2_) (b), respiratory exchange ratio (RER) (e), and total motor activity (f) in light cycle and *V*O_2_ (c), *V*CO_2_ (d), RER (g), and total motor activity (h) in dark cycle. Younger—*Tph2* ^+/+^ (*n* = 5), *Tph2* ^−/−^ (*n* = 5); Older—*Tph2* ^+/+^ (*n* = 5), *Tph2* ^−/−^ (*n* = 5). *Tph*, tryptophan hydroxylase. **p < 0.01, ***p < 0.001 and ****p < 0.0001—effect of genotype using two‐way ANOVA. ## *p* < 0.01 and ###*p* < 0.001 effect of age using Two‐way ANOVA. †*p* < 0.05 Tukey's *post hoc* test in comparison with *Tph2* ^+/+^younger mice. ‡*p* < 0.05 Tukey's *post hoc* test in comparison with Younger—*Tph2* ^+/+^. ¶*p* < 0.05 Tukey's *post hoc* test in comparison with Younger—*Tph2* ^−/−^ and Older—*Tph* ^+/+^

Aging decreased VO_2_ (light phase—*p* = 0.0008; dark phase—*p* = 0.0031) and VCO_2_ (light phase—*p* = 0.0005; dark phase—*p* = 0.0009) in both *Tph2^+/+^* and *Tph2^−/−^* mice (Figure 3). VO_2_ (light phase—*p* = 0.0004; dark phase—*p* = 0.0002) and VCO_2_ (light phase—*p* = 0.0004; dark phase—*p* < 0.0001) were significantly increased in *Tph2^−/−^* mice of both ages compared to *Tph2^+/+^* mice across the light/dark cycle (Figure 3), and no interaction with age was observed (*V*O_2_ light phase—*p* = 0.9775; *V*O_2_ dark phase—*p* = 0.3621; *V*CO_2_ light phase—*p* = 0.9270; *V*CO_2_ dark phase—*p* = 0.6078). During the light phase, aging reduced motor activity (*p* = 0.0067) and *Tph2* deficiency increased motor activity (*p* = 0.0090), while in the dark phase, only aging had a significant effect (*p* = 0.0206), and no interaction between the genotype and age was observed (light phase—*p* = 0.5859; dark phase—*p* = 0.2614).

### 3.6 The effect of *Tph2* deficiency on the respiratory equivalent (*V*_E_/*V*O_2_)

**FIGURE 4:**
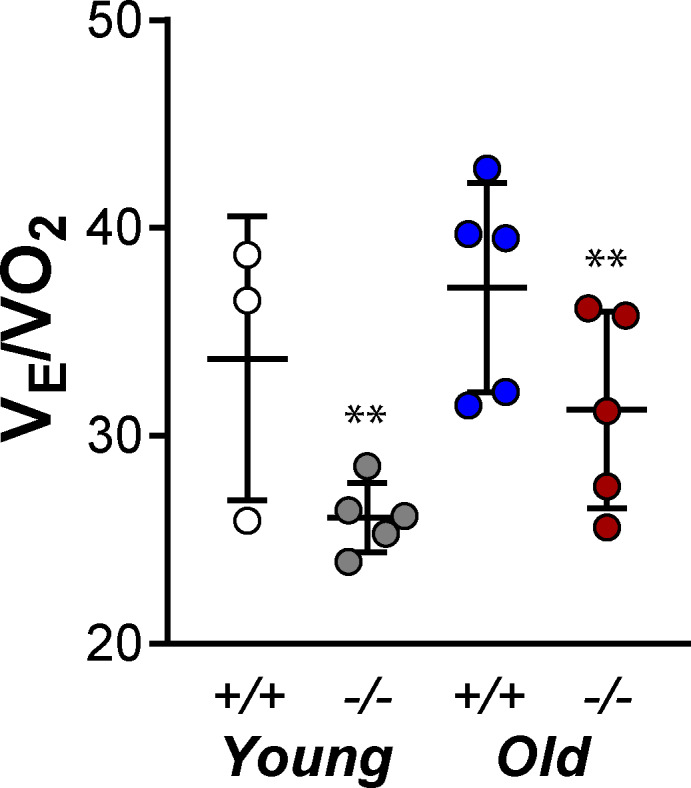
Individual and grouped data showing the age‐related differences between *Tph2* ^−/−^ and *Tph2* ^+/+^ mice on respiratory equivalent (*V*_E_/VO_2_). Younger – *Tph2* ^+/+^ (n = 3), *Tph2* ^−/−^ (n = 5); Older – *Tph2* ^+/+^ (*n* = 5), *Tph2* ^−/−^ (*n* = 5). *Tph*, tryptophan hydroxylase. **p < 0.01—effect of genotype using two‐way ANOVA

Regardless of age, *Tph2* deficiency significantly decreased *V*_E_/*V*O_2_ (*p* = 0.0088) during sleep (Figure 4). These findings suggest that *Tph2^−/−^* mice hypoventilate during sleep.

### 3.7 The effect of *Tph2* deficiency on the HCVR during wakefulness

**FIGURE 5:**
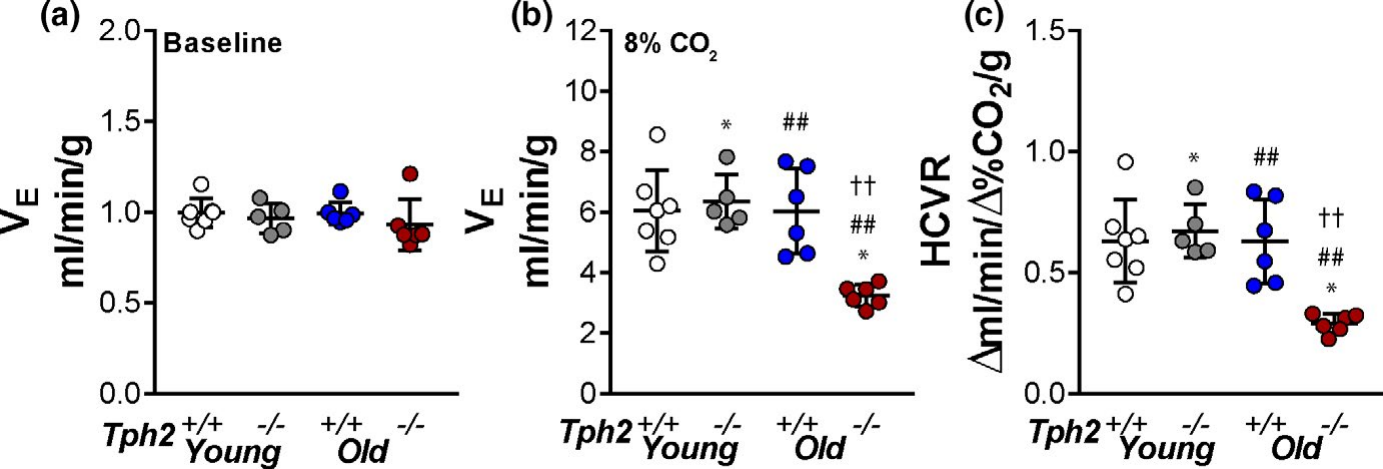
Individual and grouped data showing the age‐related differences between *Tph2* ^−/−^ and *Tph2* ^+/+^ mice one the baseline minute ventilation (*V*_E_) (a) *V*_E_ at 8% of inspired CO_2_ (b) and hypercapnic ventilatory response (HCVR) (c). Younger—*Tph2* ^+/+^ (*n* = 7), *Tph2* ^−/−^ (*n* = 5); Older – *Tph2* ^+/+^ (*n* = 6), *Tph2* ^−/−^ (*n* = 6). *Tph*, tryptophan hydroxylase. *p < 0.05—effect of genotype using two‐way ANOVA. ##*p* < 0.01 effect of age using two‐way ANOVA. ‡‡*p* < 0.01 Tukey's *post hoc* test in comparison with younger *Tph2* ^+/+^ mice and older *Tph2* ^−/^ mice

The ventilatory response to CO_2_ challenge is shown in Figure 5. During the baseline (0% of CO_2_) neither *Tph2* deficiency, aging, or a combination of the two had an effect on the *V*_E_ (*p* = 0.6763). Aging suppressed the HCVR only in *Tph2* knockout mice (*p* = 0.0020, Figure 5), but not in *Tph2^+/+^* animals. In older mice, *Tph2* deficiency dramatically decreased *V*_E_ under hypercapnic conditions (*Tph2^+/+^*: 6.03 ± 1.41 ml min^−1^ g^−1^ vs: *Tph2^−/−^:* 3.25 ± 0.36 ml min^−1^ g^−1^, *p* = 0.0017) and the HCVR (*Tph2^+/+^*: 0.63 ± 0.17 Δml min^−1^ Δ%CO_2_^−1^ g^−1^ vs *Tph2^−/−^*: 0.29 ± 0.04 Δml min^−1^ Δ%CO_2_^−1^ g^−1^, *p* = 0.0020), whereas younger mice were not affected (*p* = 0.9574). A significant interaction between aging and the genotype on hypercapnic responses was observed (*p* = 0.0032).

## 4 DISCUSSION

Our study resulted in a number of novel findings. To our knowledge, this is the first study, which examined the effect of neuronal serotonin deficiency on a combination of metabolic and respiratory parameters in the same animals, including ventilatory control, O_2_ consumption, CO_2_ production, breathing, oxyhemoglobin saturation and upper airway patency during sleep. The main finding of the study was that aging exacerbates deleterious effects of serotonin deficiency on control of breathing. Younger *Tph2* knockout animals were able to defend their breathing during hypercapnic challenge while awake, whereas older mice had depressed hypercapnic sensitivity. Sleep magnified the effects of brain serotonin deficiency on breathing across ages, and during sleep *Tph2* deficient mice were unable to mount adequate ventilatory compensation for increased metabolic rate, which resulted in hypoxemia in REM sleep. *Tph2* deficiency did not affect upper airway patency during sleep. A corollary finding of the study was that serotonin deficiency increased total sleep time and the amount of REM sleep, and decreased sleep fragmentation.

Previous studies have shown that the depletion of serotonin induces behavioral dysfunctions (Mosienko et al., 2020) and highly repetitive and compulsive behaviors (Kane et al., 2012). Mice deficient in central serotonin have impaired post‐natal growth, leading to altered autonomic control of sleep, breathing and thermoregulation (Alenina et al., 2009). Our data suggest that serotonin deficiency increases CO_2_ production. The increase in *V*CO_2_ in the serotonin deficient mice could be attributed, at least in part, to an elevated motor activity. However, the increases in metabolic activity were noted both during the dark phase, when animals are active, and during the light phase when they are predominantly asleep. In addition, *Tph2* deficient mice had elevated body temperature at rest (see Section 2) suggesting increased basal metabolic rate as a contributor to augmented CO_2_ production. Serotonin deficiency is known to increase brown adipose tissue energy expenditure (Crane et al., 2015). Notably, our current results differ from previous observations, which showed no difference in body temperature between two strains (Solarewicz et al., 2015). Hodges et al. conducted experiments in *Lmx1b^f/f/p^* with near complete absence of central 5‐HT neurons and found no effect on body temperature at an ambient temperature of 30°C, but significant hypothermia at 4°C, which they attributed to impaired shivering and non‐shivering thermogenesis (Hodges et al., 2008). These discrepancies could be attributed to the methodological and mouse strain differences. Nevertheless, our current results are consistent with previous data from our group and others showing increases in motor activity and CO_2_ production in serotonin deficiency (Carey & Kingwell, 2015; Solarewicz et al., 2015). An increase in body temperature in mice with *Tph* knockout was consistent with increased metabolism. An important novel finding of the current study is that there was no reciprocal increase in ventilation, which would allow exhalation of excess of CO_2_.

Non‐flow limited breathing is a function of metabolism and chemoreflex sensitivity to hypercapnia. The absence of a compensatory hyperventilation to increased CO_2_ production suggests that serotonin deficiency may lead to impaired hypercapnic sensitivity. In fact, our previous study showed that *Tph2^−/−^* mice have decreased sensitivity to CO_2_ during NREM sleep (Mateika et al., 2019). Similar findings were reported in mice with near complete absence of central 5‐HT neurons (Hodges et al., 2008). Our current study showed that younger *Tph2* knockout mice did not show evidence of an impaired chemoreflex during wakefulness. In contrast, older serotonin‐deficient mice consistently showed a depressed CO_2_ chemoreflex and hypoventilation. In these mice, the hypercapnic ventilatory response was severely depressed during wakefulness, which was evident from the decreased HCVR, and potentially during sleep. Although we were unable to measure the HCVR during sleep due to arousals in response to the hypercapnic stimulus, a decrease in V_I_max during non‐flow limited breathing indicates a defective HCVR (Yao et al., 2016) in *Tph2^−/−^* animals, regardless of age during sleep (Yao et al., 2016). The lack of an appropriate increase in respiration in response to metabolic demands had a more severe impact during REM sleep, the most vulnerable state. Thus, brain serotonin deficiency leads to hypoventilation during sleep, regardless of age, but younger mice with brain serotonin deficiency are able to defend their ventilation during wakefulness, whereas older mice are not.

The stimulatory effect of serotonin on the HCVR is well documented (Hodges & Richerson, 2008b). Hypercapnic sensitivity is regulated by several medullary centers, especially by the retrotrapezoid nucleus, as well as peripherally by the carotid bodies (Guyenet & Bayliss, 2015). However, serotoninergic neurons are absent at those locations. Serotonergic neurons are predominantly located in the raphe with projections to respiratory centers such as pre‐Bötzinger complex and hypoglossal motor nucleus (Ptak et al., 2009). Serotonin acts on seven subfamilies of 5‐HTR (Hilaire et al., 2010), that with the exception the 5‐HT_3_ receptor, belong to the family of seven‐transmembrane‐domain receptors that are coupled to different intracellular effectors. Subsets of 5‐HTR neurons of the raphe obscurus, a midline structure that extends throughout the medulla oblongata, is suspected to play a key role in facilitating respiratory outflow and CO_2_ responses (Depuy et al., 2011), but the molecular bases for their pH sensitivity remains unknown. The novelty of our study is that it provides the first evidence that serotonin and serotonergic respiratory neurons become more important with aging.

Neuronal serotonin deficiency had no effect on upper airway patency during REM sleep, since the frequency of inspiratory flow limitation was similar in *Tph2^+/+^* and *Tph2^−/−^* animals, regardless of age. The role of serotonin in upper airway patency during sleep is still controversial. Serotonin has been shown to increase activity of the genioglossus muscle of the tongue, a major upper airway dilator (Kubin et al., 1996). However, these experiments have been performed in anesthetized animals. Sood et al. (2005) used the intracerebroventricular micro‐dialysis technique in freely behaving unanesthetized rats found that the serotonin blocking agent 8‐OH‐DPAT inhibited genioglossus muscle activity during wakefulness, but not during sleep. Clinical studies employing serotoninergic agents were also negative (Robillard et al., 2021). To our knowledge, our study is the first in the literature to examine the effect of serotonin on upper airway patency during sleep in transgenic animals. Our data are consistent with EMG studies in sleeping animals reporting no effect of a non‐selective serotonin receptor blocker (Sood et al., 2005).

Serotonin deficiency increased sleep efficiency (Alenina et al., 2009). Here we have shown that *Tph2* deficiency consolidated sleep and increased the amount of REM sleep. Human data are consistent with experimental evidence in rodents, since selective serotonin reuptake inhibitors induce sleep fragmentation and decrease REM sleep time (Page et al., 2008; Wali & Abaalkhail, 2015).

Our study had several limitations. First, knockout of the *Tph2* gene leads to global neuronal serotonin deficiency (Alenina et al., 2009). Our findings are most likely related to serotonin deficiency in different portions of the raphe, a major source of serotonin in the brain involved in the control of breathing and upper airway function. However, serotonin may act pre‐ and post‐synaptically on multiple neurons, so it is difficult to localize the effect. Second, although we discovered that aging exacerbates the effects of serotonin deficiency on breathing, the molecular mechanisms remain obscure. Third, our study was performed in male mice and should be repeated in females to see if the effect is sex‐dependent.

In conclusion, we have shown that brain serotonin deficiency suppresses control of breathing during sleep, regardless of age. However, younger *Tph2^−/−^* animals are able to maintain the hypercapnic ventilatory response while awake, whereas older mice lose this ability. Given the ubiquitous use of selective serotonin re‐uptake inhibitors, the effect of serotonin on sleep and breathing should be considered in clinical practice.

## CONFLICT OF INTEREST

The authors declare no conflict of interest.

## ETHICAL APPROVAL

All protocols were approved by the Johns Hopkins University Animal Care and Use Committee (ACUC, Protocol #MO19M191) and all animal experiments were conducted in accordance with ACUC guidelines.

## AUTHOR CONTRIBUTIONS

Conception or design of the work (HP, MRA, QQ, MS, LJK, DMK, JHK, VYP); Acquisition, analysis, or interpretation of data for the work (HP, MRA, QQ, MS, LJK, FA, JJJ, RSA, LGSB, DMK, JHK, VYP); Drafting the work or revising it critically for important intellectual content (HP, MRA, QQ, MS, LJK, FA, JJJ, RSA, LGSB, DMK, JHK, VYP).
